# Artificial Intelligence and Psychophysiological Monitoring for Integrated Performance Modeling in Elite Soccer: A Scoping Review of Applications, Evidence Gaps, and Translational Challenges

**DOI:** 10.3390/sports14080360

**Published:** 2026-08-19

**Authors:** Ismail Dergaa, Wissem Dhahbi, Mohamed Amine Dergaa, Mortadha Razzak, Halil İbrahim Ceylan, Valentina Stefanica, Raul Ioan Muntean, Noomen Guelmami

**Affiliations:** 1 Higher Institute of Sport and Physical Education of Ksar Said, University of Manouba, Manouba 2010, Tunisia; phd.dergaa@gmail.com; 2 Physical Activity Research Unit, Sport and Health (UR18JS01), National Observatory of Sports, Tunis 1003, Tunisia; 3 Higher Institute of Sport and Physical Education of Kef, University of Jendouba, El Kef 7100, Tunisia; wissem.dhahbi@gmail.com (W.D.); amine.dergaa@gmail.com (M.A.D.); mortadhaa.razzak@gmail.com (M.R.);; 4 Physical Education and Sports Teaching Department, Faculty of Sports Sciences, Atatürk University, 25240 Erzurum, Türkiye; 5 Department of Physical Education and Sport, Faculty of Sciences, Physical Education and Informatics, Pitesti University Center, National University of Science and Technology Politehnica Bucharest, 060042 Pitesti, Romania; valentina.stefanica@upb.ro; 6 Department of Physical Education and Sport, Faculty of Law and Social Sciences, University “1 Decembrie 1918” of Alba Iulia, 510009 Alba Iulia, Romania

**Keywords:** artificial intelligence, biofeedback, decision-making, elite soccer, explainable AI, heart rate variability, machine learning, performance modeling, psychophysiology, scoping review, wearable sensors

## Abstract

Background: Elite soccer performance emerges from the interplay of cognitive, emotional, psychophysiological, and tactical processes that operate in real time during matches. Advances in wearable sensors and artificial intelligence (AI) now allow continuous monitoring of physiological and psychological states. They also allow modeling of how these states relate to tactical and physical performance. Existing reviews have examined machine learning in soccer, heart rate variability (HRV) monitoring, and psychological determinants of performance separately. No scoping review has mapped the intersection of AI analytics, wearable psychophysiological monitoring, and psychological performance constructs as one integrated decision-support framework in elite soccer. Aim: The aim of this study was to map the available evidence on the integration of AI and machine learning with psychophysiological monitoring for performance modeling in elite soccer, to identify the psychological constructs already used as model inputs, to describe the wearable technologies and AI methods applied, and to set out the translational challenges and evidence gaps that need priority attention. Methods: The review followed the PRISMA extension for Scoping Reviews (PRISMA-ScR) and the updated Joanna Briggs Institute (JBI) methodology. The protocol was registered on the Open Science Framework (OSF). Six databases (PubMed/MEDLINE, Scopus, Web of Science, SPORTDiscus, IEEE Xplore, and PsycINFO) were searched from January 2000 to March 2026 using the Population–Concept–Context (PCC) framework. Two reviewers independently screened titles, abstracts, and full texts (Cohen’s kappa = 0.82). Results: Thirty-six sources met the eligibility criteria after screening of 3104 records. AI and machine learning have been applied widely to predict physical and tactical performance in soccer, yet they rarely include psychological constructs. Reported models (decision trees, gradient boosting, and artificial neural networks) reach high accuracy for physical outcomes in internal validation, for example, above 66% for injury risk. Multi-modal models that add physiological and psychological inputs report stronger prediction. These figures come mostly from internal validation, and external validation and overfitting controls are seldom reported, so they should be read as optimistic upper bounds. Psychological and psychophysiological inputs remain under-represented. Explainable AI (XAI) methods, in particular Shapley Addictive exPlanations (SHAP) values, are appearing, but validation with domain experts is scarce. HRV has been reviewed as a psychophysiological marker in soccer, yet its use within AI decision-support tools for real-time psychological readiness has not been mapped. Three translational challenges stand out: the ecological validity gap between laboratory cognitive tests and match-embedded psychophysiology; the interpretability problem of opaque AI in high-stakes decisions; and the data fragmentation problem created by disconnected physical, tactical, and psychological data streams. Conclusions: Integrating AI with wearable psychophysiological monitoring offers a credible route toward integrated performance modeling in elite soccer. Closing this gap calls for multi-modal frameworks that combine psychological constructs, physiological markers, and tactical data within explainable AI. Research priorities include ecologically valid psychophysiological assessment protocols, position-specific psychological profiling, and practitioner-validated tools that turn AI outputs into usable coaching recommendations.

## 1. Introduction

Elite soccer is among the most cognitively and physiologically demanding settings in competitive sport. Match performance depends on physical fitness and technical–tactical skill. It also depends on the continuous, real-time use of attentional control, decision-making under pressure, emotional regulation, and psychophysiological readiness [1,2]. The review by Stølen et al. [1], still the most cited analysis of soccer physiology with roughly 5000 citations, covered physical and physiological parameters in depth. It could not anticipate the change in measurement and modeling capacity of the two decades since. Today, global positioning system (GPS) tracking, accelerometry, heart rate telemetry, salivary biomarker sampling, and wearable electrodermal sensors produce continuous multi-dimensional data from every session and match. The analytical infrastructure needed to combine these streams with the psychological constructs that shape performance under pressure did not exist in 2005. It exists now, and the field is only just starting to use it.

Machine learning (ML) and AI have been applied to soccer data in three main areas: injury prediction, physical and physiological performance forecasting, and tactical analysis [3,4,5]. Systematic reviews report that ML models predict injury risk from pre-season screening with an accuracy above 66% [4]. They forecast match outcomes with high sensitivity from positional and event data [6]. They also identify tactical signatures of defensive success through explainable AI [7]. The scoping review by Munoz-Macho et al. [5] found 32 studies using AI in elite team sport, of which 67% involved soccer, with tree-based algorithms most common. A 2025 systematic review of 172 ML papers in professional soccer confirmed the dominance of supervised learning and deep learning for physical performance and match-outcome prediction [8]. Together, these reviews show that AI has reached technical maturity for processing physical and tactical data at scale in soccer.

Alongside these advances, the sport psychology literature has described the psychological determinants of elite soccer performance with growing precision. Decision-making under pressure depends on executive functions, including inhibitory control, working memory, and cognitive flexibility. Elite players show systematically stronger cognitive profiles than sub-elite athletes [2,9,10]. Psychophysiological stress responses, measured through HRV, salivary cortisol, and electrodermal activity, change executive function in game-realistic conditions. Psychological stress induction produces measurable changes in inhibitory control and cognitive flexibility in elite youth players [11]. The general cognitive model proposed by Habekost et al. [2] links situational assessment, action selection, and outcome evaluation through a perception–action loop that psychological readiness and emotional state regulate. Bonetti et al. [9] used artificial neural networks to show that elite players have a distinct psychological profile, marked by higher cognitive flexibility, higher conscientiousness, and lower neuroticism, and that this profile relates to competitive standing.

The main gap in the literature is the absence of work that connects these two advancing fields. AI reviews of soccer do not use psychological constructs as model inputs [3,4,5,6,8]. Reviews of psychophysiological monitoring in soccer treat HRV as a physiological marker of training load and recovery. They do not examine AI-driven integration with decision-making, emotion regulation, or wider psychological constructs [12,13,14]. Reviews of wearable biosensors fused with AI for mental health monitoring show that continuous psychological-state assessment is feasible [15,16], but these approaches have not been applied to soccer. The sport psychology AI literature names the under-representation of psychological constructs in data-driven talent identification and performance models as a priority [17]. No review has yet mapped what has been done and what remains to be done at this intersection.

This scoping review was guided by four research questions, each linked to a results section: RQ1: What is the volume, study design, and geographic distribution of research on AI and psychophysiological monitoring in elite soccer from January 2000 to March 2026 (Section 3.1, Section 3.2 and Section 3.3)? RQ2: Which psychological and psychophysiological constructs have been used as inputs in AI-driven performance models (Section 3.4)? RQ3: Which wearable technologies and AI methods have been applied, and what does the evidence outside soccer show about feasibility (Section 3.4 and Section 3.5)? RQ4: What translational challenges and priority evidence gaps stand between current knowledge and ecologically valid, practitioner-facing decision-support tools (Section 3.6)? These questions map directly onto the four review objectives and onto the structure of the Results and Discussion sections.

## 2. Materials and Methods

### 2.1. Study Design and Registration

This scoping review was conducted and reported in line with the PRISMA extension for Scoping Reviews (PRISMA-ScR) [18], which extends the 2020 PRISMA statement to the features of scoping reviews. The 22-item PRISMA-ScR checklist guided reporting of all review components, including the rationale for the scoping approach, the eligibility criteria framed through the PCC framework, the information sources and search strategies, the selection and data-charting processes, and the synthesis methods. The completed checklist is provided as Supplementary Material S1. The protocol was registered on the Open Science Framework (OSF; https://osf.io/ad957/; registration identifier: https://doi.org/10.17605/OSF.IO/AD957) on 22 May 2026. PROSPERO does not accept scoping review protocols, so OSF was used for registration.

The scoping review method was chosen over a systematic review with meta-analysis for a specific reason. The aim is to map the breadth and distribution of an evidence landscape that spans several fields, namely, artificial intelligence, wearable sensor technology, exercise physiology, and sport psychology. The aim is not to produce a pooled quantitative estimate from a homogeneous body of trials. This choice follows JBI guidance for scoping reviews [19], which recommends the scoping method when the field is heterogeneous, when a preliminary evidence map is needed, and when formal critical appraisal and evidence grading are not the main purpose.

**Figure 1:**
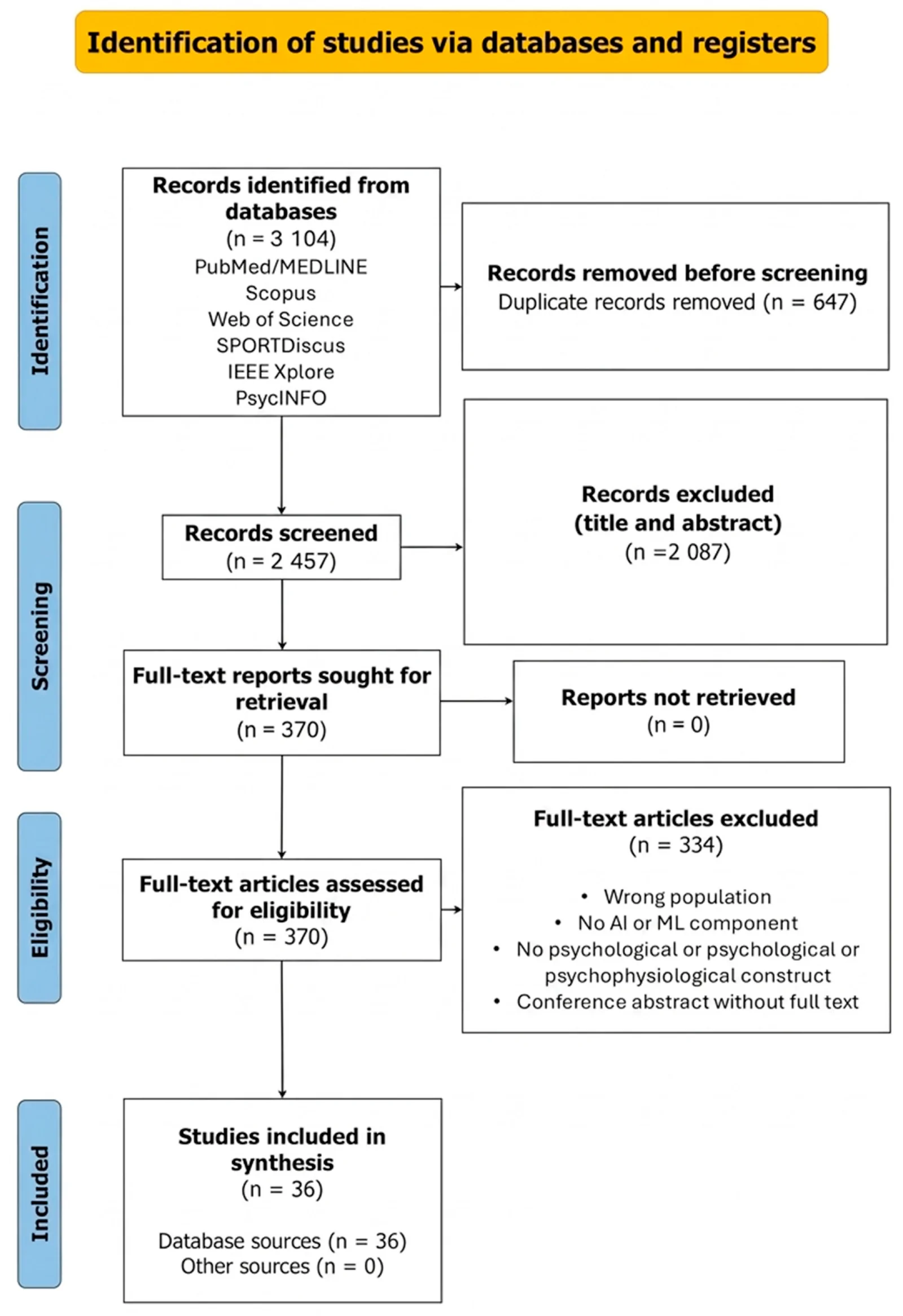
PRISMA-ScR flow diagram of identification, screening, and selection. Six databases were searched for January 2000 to March 2026. Records identified: 3104; duplicates removed: 647; records screened: 2457; excluded at title-and-abstract stage: 2087; full texts assessed: 370; full texts excluded: 334 (reasons: wrong population; no AI or ML component; no psychological or psychophysiological construct; conference abstract without full text). Sources included: 36.

In line with the JBI scoping review methodology [19], formal critical appraisal of individual sources was not performed. Two reviewers independently screened all records at the title-and-abstract stage and reached strong agreement (Cohen’s kappa = 0.82). Disagreements were resolved by consensus, and a third author arbitrated unresolved cases. The selection process is shown in the PRISMA-ScR flow diagram (Figure 1).

### 2.2. Eligibility Criteria

Eligibility was defined using the Population–Concept–Context (PCC) framework recommended for scoping reviews [19]. The full criteria appear in Table 1. The review deliberately included two levels of evidence: primary studies and evidence syntheses (systematic, scoping, and narrative reviews). This decision reflects the early stage of the field. Reviews give landscape coverage of mature sub-areas such as ML in soccer and HRV monitoring, while primary studies give the direct empirical findings on psychological and psychophysiological inputs. The publication-type row in Table 1 states this inclusion explicitly. To prevent double counting, each source was charted at its own evidence level, and any overlap between a review and its constituent primary studies was flagged during charting. When a primary study and a review that included it both appeared, quantitative claims were attributed to the primary source, and the review was retained only for its synthesis-level mapping.

### 2.3. Information Sources and Search Strategy

Six databases were searched: PubMed/MEDLINE, Scopus, Web of Science, SPORTDiscus, IEEE Xplore, and PsycINFO. The period from January 2000 to March 2026 was chosen to capture the full arc of ML in soccer, from early computational performance analysis to the current generative AI era. Reference lists of all included full-text sources were hand-searched. The search used the same three concept blocks across every database: a soccer block, an AI and machine learning block (including deep learning, neural networks, and explainable AI), and a psychophysiological and psychological block (including HRV, psychophysiology, decision-making, executive function, emotion regulation, mental toughness, cognitive performance, wearable, biosensor, and cortisol). The same terms were used for each concept block in every database string; only field-restriction tags, truncation symbols, and Boolean syntax were adapted to each platform’s search interface, so the conceptual scope of the search stayed identical across sources. The complete strings appear in Table 2.

### 2.4. Selection Process

Retrieved records were imported into Covidence for deduplication and screening. Two independent reviewers (I.D. and N.G.) screened all titles and abstracts against the PCC criteria. Agreement at this stage was strong (Cohen’s kappa = 0.82). Disagreements were resolved by consensus, and W.D. arbitrated unresolved cases. Full-text assessment was performed for every record that passed initial screening. Reasons for exclusion at the full-text stage were recorded for each excluded source. The PRISMA-ScR flow diagram is presented in Figure 1 in the main text.

### 2.5. Data Charting and Synthesis

Data were charted with a standardized form piloted on eight randomly selected sources before full use. The charted variables were: first author, year, country of origin, evidence type (primary study or review), study design, player population (level, sex, and age), AI or ML method, psychological or psychophysiological constructs assessed, wearable technologies used, key quantitative findings, translational limitations, and conclusions. Charting was done independently by I.D. and M.A.D., and W.D. resolved discrepancies. The charting form and the full extraction table are provided as Supplementary Material S2. Descriptive synthesis characterized the volume and distribution of the evidence. Thematic synthesis organized findings across four domains: AI and physical–tactical performance modeling, psychophysiology and psychological constructs in soccer, AI-integrated psychophysiological monitoring, and translational challenges and evidence gaps.

Because the included evidence was heterogeneous, conclusions were weighted by evidence type rather than treated as equivalent. Empirical primary studies carried the most weight for claims about effects and accuracy. Reviews were used to map the landscape of mature sub-areas. Opinion and theoretical papers were used only for framing, and statements drawn from them are identified as conceptual rather than empirical throughout the text. No meta-analysis was conducted, given the heterogeneity of designs and outcomes, and formal critical appraisal was not performed, consistent with the JBI scoping methodology [19].

### 2.6. AI Usage Statement

Claude (Anthropic) assisted with reference organization and structural drafting during manuscript preparation. The lead author (I.D.) conceived the review, performed the literature search, led data charting, wrote and critically revised all sections, and takes full scientific and ethical responsibility for the content. All AI-assisted text was reviewed, verified, and substantially revised by the author team before submission.

## 3. Results

### 3.1. Study Selection

The six-database search retrieved 3104 records. After removal of 647 duplicates, 2457 unique records were screened at the title and abstract stage. Of these, 2087 were excluded for not addressing the intersection of AI, psychophysiology, or psychological constructs in soccer. The remaining 370 full-text articles were assessed for eligibility. Of these, 334 were excluded for wrong population, no AI or ML component, no psychological or psychophysiological construct, or absence of full text. The final corpus comprised 36 charted sources. Figure 1 presents the flow diagram. The full list of these 36 sources, with evidence type and charted characteristics, is provided as Supplementary Material S2, which separates the included evidence base from the methodological and foundational works cited only for background.

### 3.2. Characteristics of Included Sources

The 36 included sources span 2014 to March 2026. Publication volume rose sharply from 2024 onward: one source between 2010 and 2018, eight between 2019 and 2023, and 27 in 2024 to 2026. The bands are unequal by design. They follow recognized inflection points in the field rather than fixed decades. The 2010 to 2018 band covers sensor adoption and early ML. The 2019 to 2023 band covers the expansion of deep learning. The 2024 to 2026 band covers the arrival of explainable and generative AI. Grouping by these milestones makes the growth pattern easier to read than equal intervals would. The sources originated from research groups in several countries. By evidence type, the corpus comprised 25 reviews (systematic, scoping, narrative, and meta-analytic), eight primary experimental or observational studies, two conceptual or opinion papers, and one bibliometric analysis. Table 3 shows the distribution across thematic domains with principal methods and key findings.

### 3.3. AI and Machine Learning in Soccer: Physical–Tactical Modeling Landscape

The most fully mapped area is the application of ML to physical and tactical data in professional soccer. The 2023 systematic review by Rico-González et al. [4] found 32 studies clustering into injury prediction, physical and physiological forecasting, and technical-tactical forecasting, with decision trees the most frequent algorithm. Injury prediction models built from pre-season screening, training load, and neuromuscular survey data reached accuracy above 66% with decision-tree classifiers [4]. Physical performance models that used GPS external-load metrics and internal-load ratings estimated wellness and fatigue indices that could inform training-load adjustments [4]. These accuracy values come mainly from internal validation. External validation across independent squads and seasons and explicit checks for overfitting are rarely reported, so the figures should be read as upper bounds rather than field-ready performance. The 2024 systematic review by Elstak et al. [25] raised the same concern about the opaque nature of deep learning in soccer, noting that high accuracy does not bring the interpretability that high-stakes performance and medical decisions require.

The 2025 systematic review of 172 ML papers in professional soccer [8] confirmed the dominance of supervised learning and hybrid deep learning. Across applications, models were trained mostly on physical and event data; psychological and psychophysiological inputs appeared in fewer than 8% of the included models [8]. The scoping review by Munoz-Macho et al. [5] identified soccer as the most studied sport in AI elite team-sport research (67% of 32 studies), with healthcare applications slightly more common than performance applications. As a primary application study, Forcher et al. [7] showed that tracking data combined with explainable AI (gradient boosting with SHAP) could identify the tactical signatures of defensive success in elite soccer with high ecological validity. This is the most mature example of XAI applied to match performance data. None of these models, however, used psychological readiness, emotional state, or psychophysiological markers alongside the physical and tactical inputs.

The dedicated scoping review of XAI in sports science [26], covering 19 studies from 2014 to June 2024, confirmed that SHAP values are the dominant XAI approach across sports and found a complete absence of psychological constructs in input feature sets. The review flagged the lack of domain-expert validation of AI explanations as a critical gap. SHAP outputs produced for coaches and performance staff have rarely been tested for comprehensibility or usefulness in real coaching settings [26]. This gap matters for the integration of psychological data into AI. Without explanation methods that turn multi-dimensional psychophysiological data into coaching-ready information, even accurate models will not be adopted by practitioners.

### 3.4. Psychological Constructs and Psychophysiological Monitoring in Elite Soccer

#### 3.4.1. Cognitive Performance and Executive Function

The scoping review by Haugan et al. [10], covering 15 empirical studies published between 2000 and 2023, established that executive functions, specifically inhibitory control, working memory, and cognitive flexibility, are positively associated with elite soccer performance. The same review noted that the ecological validity of laboratory cognitive tests remains a limitation. The general cognitive model [2] proposes three functional stages: situational assessment through perceptual–attentional processing; action selection through decision-making under time pressure; and outcome assessment through error detection and recalibration. Each stage recruits distinct cognitive resources. These can be measured with standardized neuropsychological batteries and, in principle, quantified with wearable neurophysiological tools in the field.

As the strongest primary study at this intersection, Bonetti et al. [9] applied artificial neural networks and support vector machines to 328 participants, including 204 elite players from top-tier Brazilian and Swedish teams. The neural-network model decoded the psychological profile of elite players, identifying higher cognitive flexibility, planning, and memory, together with higher conscientiousness and openness, as distinguishing features. This is the highest-quality integration of AI with psychological profiling in soccer to date. It relied on off-field laboratory assessment, however, rather than in-match psychophysiological monitoring [9]. The perceptual–cognitive training meta-analysis by Zhu et al. [29], a review-level source, reported a larger effect size (ES) for laboratory training (ES = 1.51) than for transfer to real-game performance (ES = 0.65). This contrast restates the ecological validity challenge that any integrated monitoring framework must address.

#### 3.4.2. Psychophysiological Stress and Performance Under Pressure

The experimental study by Knöbel et al. [11] showed that soccer-specific psychophysiological stress induction, combining physical stress with competitive instructions, produced significant changes in objective markers (cortisol and HRV) and subjective markers in 92 elite youth players. No significant interaction between condition and cognitive performance appeared at the group level. Descriptive statistics, however, suggested better performance under stress in some executive-function domains, and response-time variability fell under the stress condition. This pattern fits the challenge–threat literature, which holds that stress can help performance when athletes appraise it as a challenge rather than a threat and that elite players may develop stress-adapted processing through years of high-pressure matches. The methodological implication for integrated monitoring is direct. Psychophysiological stress markers and cognitive performance should be measured at the same time, in ecologically valid conditions, to capture these context-dependent interactions [30].

Pre-competition psychophysiological states in elite players involve raised cortisol relative to non-training baselines, together with mostly pleasant affective states in starters and more variable profiles in non-starters [31]. This difference according to competitive status has a clear implication for AI-driven readiness monitoring. A model that treats all squad players as equivalent will misestimate the readiness of those outside the starting line-up, the group most exposed to motivational and attentional dysregulation during matches [31]. Position-specific and status-specific psychophysiological profiling is a high-priority direction that no current AI performance model has put into practice.

#### 3.4.3. HRV as a Connecting Psychophysiological Marker

HRV holds a distinctive place in this landscape as the most mature psychophysiological marker for field-based monitoring in soccer. The scoping review by Mirto et al. [13], covering 25 studies in professional and semi-professional soccer, confirmed that morning vagally mediated HRV determined using ultra-short orthostatic measurements is an ecologically feasible way to assess training adaptation and readiness. The systematic review by Lipka et al. [14], covering 19 studies, confirmed that HRV indices, in particular the time-domain parameter RMSSD, relate to overtraining symptoms, psychological aspects of fatigue, and physiological recovery in soccer players. The meta-analysis by García-Ortega et al. [12] found a small, non-significant effect of soccer training on HRV at the group level (ES = 0.143). It found larger, significant effects for female players (*p* = 0.027, d = 0.275) and professional players (*p* = 0.007, d = 0.302). These contrasts support individual and contextual interpretation rather than group averages.

None of these reviews addresses the AI-driven integration of HRV with cognitive and emotional performance data in a real-time decision-support context. The bibliometric analysis of HRV in sports science from 2010 to 2025 [32] identified ML-guided HRV interpretation as an emerging frontier and flagged it as underexplored. In a primary study, Li et al. [33] reported that pre-sleep HRV predicted chronic insomnia in national-level athletes with 96% accuracy using binary logistic regression. This figure comes from a single sample without external validation, so it shows proof of concept rather than established accuracy. It does show that even one physiological signal, processed through a simple model, can yield useful predictions when the outcome is well defined. Translating this proof of concept to real-time psychological readiness in competitive soccer, by combining RMSSD trends with psychological questionnaires, training load, and match context, is the most technically proximate next step for applied psychophysiological AI in this sport.

### 3.5. AI-Integrated Psychophysiological Monitoring: Current Evidence and Feasibility

The evidence most relevant to the proposed integration comes from research conducted outside soccer. These sources were eligible under the PCC clause that admits laboratory and adjacent-population studies directly translatable to field application, and they are charted as contextual feasibility evidence rather than soccer-specific findings (Supplementary Material S2). They are reported here to establish technical feasibility, and they are kept distinct from the soccer-specific evidence above. A 2025 systematic review of wearable biosensors fused with AI for mental health monitoring [16], covering 48 studies that used HRV, electrodermal activity (EDA), and accelerometry with AI, reported that ML models trained on wearable data can classify stress (about 60% of studies), depression (about 31%), and anxiety (about 9%) with meaningful accuracy. The most consistent finding was that multi-modal sensor fusion, combining HRV, EDA, and motion data, outperformed single-signal models for psychological-state classification [16]. A parallel systematic review of wearable physiological monitoring in exercise and mental health [15] confirmed that HRV, electrodermal activity, and respiration are the most informative signals for detecting acute psychological-state changes during and after exercise.

A multi-modal ML framework that combined physiological signals (HRV, oxygen consumption, and muscle activation) with psychological inputs (mental toughness, athlete engagement, and team cohesion) in 480 athletes from several sports reported a correlation of R = 0.90 between predicted and observed performance, above the R = 0.77 reported for unidimensional physical or statistical models [24]. This study, although not soccer-specific, gives the strongest available empirical support for the central premise of this review: that combining physiological and psychological data within AI architectures improves performance prediction relative to physical data alone. The soccer-specific version of this framework, with match-embedded psychophysiological monitoring through validated wearables alongside AI analytics, remains an open research territory. This last statement is a conceptual projection from adjacent evidence, not a description of existing soccer practice.

Biofeedback and neurofeedback represent the most mature route from psychophysiological monitoring to performance intervention. A 2025 Bayesian meta-analysis [34] reported that HRV biofeedback produces moderate effects for anxiety reduction and performance, operating through better self-regulation and interoceptive awareness. The 2020 systematic review and meta-analysis by Lehrer et al. [35] found significant small-to-moderate effects of HRV biofeedback across domains, with the largest effects for anxiety and athletic or artistic performance. Neurofeedback training produces measurable gains in reaction time, attentional control, and cognitive performance in athletes [28], and a biofeedback intervention in soccer players reported direct performance benefits [34]. These findings show that the physiological basis for closed-loop psychophysiological intervention already exists. The missing element is the AI layer that would individualize, automate, and scale these interventions from isolated laboratory protocols to routine practice.

### 3.6. Translational Challenges: Three Priority Gaps

#### 3.6.1. The Ecological Validity Gap

The most basic translational challenge is the ecological validity gap between laboratory cognitive tests and the psychophysiological demands of competitive play. Laboratory executive-function tests such as the Stroop task, the Trail Making Test, and the flanker paradigm measure executive functions in static, decontextualized conditions. These conditions share only part of their variance with the real-time decision-making of a 90 min match [2,10]. The perceptual–cognitive training meta-analysis confirmed that the effect size for real-game transfer (ES = 0.65) is much smaller than for laboratory improvement (ES = 1.51) [29]. Knöbel et al. [11] tried to close this gap by running executive-function tests under game-like stress, yet even that design could not fully reproduce the proprioceptive, social, and motivational context of competition.

Closing this gap needs three concurrent advances: first, validated match-embedded psychophysiological assessment protocols that use wearables and do not interfere with performance; second, AI models trained on ecologically valid in-situ data rather than laboratory proxies; third, the inclusion of practitioner knowledge about game context, scoreline, opponent quality, and individual history within AI interpretive frameworks [17,30]. The field’s emphasis on ecological validity and representative design reflects a shared view that this gap is the main barrier to translation, not a secondary refinement.

#### 3.6.2. The Interpretability Problem

The second gap is the interpretability problem: the mismatch between the technical outputs of AI models and the working language of coaching and performance staff. Deep learning models trained on multi-modal psychophysiological and tracking data may reach high accuracy for readiness while producing outputs that a coach preparing a match-day squad cannot use. The XAI scoping review [26] named the lack of domain-expert validation of AI explanations as the most critical gap in sports science XAI, noting that SHAP outputs for coaches have rarely been tested for comprehensibility or usefulness. The football-codes AI systematic review [25] raised the same concern and framed explainability as an ethical obligation when AI informs high-stakes decisions such as selection, injury clearance, or return-to-competition.

The psychological domain makes this problem sharper because the constructs at stake, such as emotional state, motivational readiness, and threat-versus-challenge appraisal, are subjective and context-bound [30]. A SHAP value showing that an HRV reduction over three training days drives a model’s prediction of lower decision-making accuracy carries clinical meaning only when the coach can place it within the player’s recent travel, emotional state, and positional demands. The central design task for the next generation of integrated monitoring tools is to build AI systems that communicate uncertainty, surface relevant context, and produce recommendations in the everyday language of soccer practitioners [7,17,26].

#### 3.6.3. The Data Fragmentation Problem

The third challenge is data fragmentation: the separation of physical, tactical, physiological, and psychological data into isolated silos that block integrated modeling. Physical external-load data usually sit in GPS software platforms (such as Catapult and STATSports). Tactical tracking data sit in specialist analytics platforms (such as Opta, StatsBomb, and Wyscout). HRV and recovery data sit in physiological monitoring apps. Psychological data sit in questionnaire platforms or standalone tools. These streams are rarely linked at the individual player level and almost never fused in real time [5,8].

Multi-modal integration is technically feasible. As a primary study, Jianjun et al. [24] showed that physical and psychological data can be fused within gradient boosting and neural-network frameworks, with clear accuracy gains. The recovery-prediction study in youth soccer [36], which combined GPS external, internal, and cognitive–tactical indicators within a supervised ML framework, confirmed that multi-dimensional fusion outperforms single-domain models even in sub-elite groups. The barrier to routine use in professional soccer is organizational and ethical rather than technical. Data governance frameworks, consent procedures, player rights over biometric and psychological data, and interoperability standards across platforms all need to be settled before integrated monitoring becomes routine [25,30]. The field is calling for standardized multi-modal data-collection protocols and open data-sharing agreements as preconditions for the next stage of integrated performance research [6,25].

## 4. Discussion

This scoping review mapped the evidence at the intersection of AI, wearable psychophysiological monitoring, and psychological performance constructs in elite soccer, across 36 sources and more than a decade. The central finding is that two major frontiers, AI-driven performance modeling and psychological performance science, have developed almost in parallel without real integration. AI models for soccer performance are technically sophisticated but psychologically thin. Psychological performance research in soccer is theoretically rich but computationally underpowered. The integrated framework that motivated this review has been described in concept but not built in practice. Three themes deserve emphasis. The forward-looking statements in this section are presented as research directions supported by adjacent evidence, not as established findings in soccer.

### 4.1. The Convergence Opportunity: HRV as a Psychological Performance Marker

HRV connects the AI–physical performance literature and the psychological performance literature. It is at once a physiological training-load marker [13,14], a readiness indicator [12,37,38], a psychological stress marker [11,30,33], and a modifiable target through biofeedback that improves both self-regulation and performance [27,35]. Pre-sleep HRV predicted insomnia in team-sport athletes with 96% accuracy in a single sample [33]. Morning RMSSD trends across a training week capture functional overreaching before performance drops become clinically clear [37]. Pre-competition cortisol–HRV coupling separates starter from non-starter emotional states in elite soccer [31]. The mapping of HRV in sports science from 2010 to 2025 [32] confirms that ML-guided HRV interpretation is an identified emerging frontier. What is missing is the integration of HRV-based readiness signals with cognitive performance measures and AI decision-support in soccer-specific contexts.

The practical route to this integration is now accessible. Validated short-recording wearables that measure RMSSD with accuracy close to the electrocardiography (ECG) reference are commercially available and already used in professional clubs. ML algorithms trained on individual HRV baselines, adjusted for training load, sleep, and match schedule, can in principle generate daily readiness profiles that reflect both physiological recovery and psychological stress. Adding brief validated self-report instruments, delivered through club monitoring apps, would calibrate physiological signals against subjective state and produce richer composite readiness indices than either source alone. The theoretical basis for this integration is the biopsychosocial framework of Kabeer Dilshith et al. [30], which reframes stress in sport as a multisystem regulation process that requires simultaneous physiological, psychological, and contextual assessment.

### 4.2. The Psychological Profile as an AI Input Layer

The study by Bonetti et al. [9] is a proof of concept for AI-driven psychological profiling in elite soccer, and it also shows the limits of the current approach. It is a cross-sectional, laboratory-based assessment of personality and cognitive traits that identifies who elite players are, not how their psychological state changes across a season. The more operationally relevant task for integrated AI models is to capture within-player variation in readiness across the training cycle, the congested schedule, international travel, injury recovery, and competitive pressure. This requires longitudinal multi-modal data rather than cross-sectional profiling. It also requires models that learn individual baseline profiles and detect meaningful deviations from them [17,23].

The multi-modal model of Jianjun et al. [24] reported a correlation of R = 0.90 when psychological inputs were added to physiological data, compared with R = 0.77 for physiological or statistical models alone. This gives a quantitative argument for including psychological constructs in soccer performance models. The increment is not trivial in a sport where small differences decide matches and careers, although it comes from a multi-sport sample and needs replication in soccer with external validation. The finding aligns with sport psychology evidence that psychological factors, including decision-making, hope for success, and task orientation, have small but significant prospective relationships with future soccer performance [39]. The task is to build AI architectures that capture these relationships with ecological validity across a full season at squad scale.

### 4.3. Practitioner-Centered Design as a Requirement

The most consistent finding across the translational literature is that AI systems in elite sport are not adopted when their outputs are not built around the decision-making language and workflow of coaches and performance staff [25,26,30]. The XAI scoping review [26] names the lack of domain-expert validation as the defining gap. The emphasis on practitioner–researcher collaboration and ecological validity is a requirement, not an optional refinement. A perfectly accurate model for psychological readiness that outputs SHAP values referencing RMSSD z-scores, cortisol-to-dehydroepiandrosterone (DHEA) ratios, and inhibitory-control latency will be overridden by the coach’s impression of the player in training, because the model speaks a language foreign to the coaching environment.

Practitioner-centered design requires iterative co-development between sport scientists, AI developers, and coaching staff across the whole model cycle, not just at the output stage. It requires that the psychological and psychophysiological constructs in the model are recognized by coaches as meaningful performance determinants, not merely significant inputs. It requires that uncertainty is communicated plainly rather than buried in confidence-interval notation. It also requires that outputs produce recommendations that fit existing workflows rather than demanding new data-collection behaviors that compete with session time and player trust. The implication for research design is clear. The next generation of integrated performance studies in elite soccer should embed practitioner co-design from the first conceptual stage.

### 4.4. Future Research Priorities

Five priorities follow from this review: first, prospective longitudinal studies that embed wearable HRV and EDA monitoring in professional squads across full seasons to establish individual baseline profiles and their relationships with match-day outcomes; second, validated match-embedded assessment protocols that measure cognitive and emotional indicators in ecologically valid conditions without interfering with play, combining brief digital self-report, HRV monitoring, and post-match biometric analysis; third, AI models that integrate physical, tactical, and psychophysiological data within explainable architectures validated with coaching staff, tested against single-domain models to quantify the added value of psychological inputs; fourth, position-specific psychological profiling within integrated monitoring frameworks, given documented differences in executive-function demand, emotional regulation, and stress profiles across positions; fifth, international consensus guidance for multi-modal data collection, storage, and governance in professional soccer to enable the data-sharing infrastructure that integrated AI modeling needs.

### 4.5. Limitations

This review has several limitations. Restricting publications to English introduces a language bias that may exclude relevant work from research communities, including those in South America and East Asia, where soccer AI research is active. The included sources are heterogeneous, spanning laboratory experiments, observational cohorts, reviews, and opinion papers, so evidence quality is not uniform, and the scoping method does not weight sources by design quality. Both the AI methods literature and the wearable sensor field move quickly, so some findings may be superseded between the search date and publication. The lower boundary of January 2000 captures the ML application literature from its emergence in sports science, but it excludes earlier psychophysiological research that gives foundational context for some constructs. Finally, because no integrated AI–psychophysiological monitoring framework yet exists in elite soccer, the most applied parts of this synthesis are projective. They extrapolate from feasibility evidence in adjacent populations rather than describe established practice in the target setting, and they are labeled as such in the text.

## 5. Conclusions

This scoping review mapped 36 sources across more than a decade at the intersection of artificial intelligence, wearable psychophysiological monitoring, and psychological performance constructs in elite soccer. The evidence shows that AI and machine learning have reached technical maturity for physical–tactical performance modeling in soccer, yet they have not incorporated the psychological and psychophysiological constructs that the sport psychology literature identifies as central to competitive success. HRV is the most mature psychophysiological marker for field-based monitoring, but its integration with AI decision-support for psychological readiness has not been charted. Three translational challenges, the ecological validity gap, the interpretability problem, and the data fragmentation problem, are the main barriers between the current evidence and practitioner-facing tools. Addressing them requires ecologically valid in situ assessment protocols, explainable AI architectures validated with practitioners, and multi-modal data-governance frameworks that link physical, tactical, and psychophysiological streams at the individual level across full seasons. The integrated performance-modeling framework described here is technically achievable. Reaching it depends on the kind of interdisciplinary practitioner–researcher collaboration that this work is intended to support.

## Figures and Tables

**Table 1:** Eligibility criteria using the Population–Concept–Context (PCC) framework.

Component	Inclusion Criteria	Exclusion Criteria
Population	Elite and semi-professional soccer players (male or female, adult); coaching and performance staff in professional soccer; youth elite academy players	Recreational, amateur, or non-athletic populations; elite athletes in non-soccer sports, except where comparative evidence is directly relevant to methodology
Concept	(1) AI, machine learning, or deep learning applied to performance modeling or psychophysiological monitoring; (2) psychological constructs such as executive function, decision-making, emotion regulation, mental toughness, attentional control, and resilience; (3) wearable physiological sensors measuring autonomic, neuroendocrine, or neurophysiological parameters; (4) integration of two or more of the above in a soccer context	Studies addressing only tactical or physical performance with no psychological or psychophysiological component; studies using AI only for video analysis with no physiological or psychological data
Context	Training, match-day, pre-competition, and recovery contexts in professional or elite soccer; laboratory studies directly translatable to field application, charted as contextual feasibility evidence	Studies conducted only in recreational sport or non-competitive physical activity
Publication	English-language peer-reviewed work, including both primary studies and evidence syntheses: original experimental studies, observational studies, systematic reviews, meta-analyses, scoping reviews, and narrative or opinion papers with empirical grounding; published January 2000 to March 2026	Conference abstracts without full text; gray literature without peer review; letters or editorials without empirical content

**Table 2:** Search strings applied across six electronic databases. The three concept blocks (soccer; AI and machine learning; psychophysiological and psychological) were held constant; only platform syntax was adapted.

Database	Search String
PubMed/MEDLINE	((soccer OR football) AND (“machine learning” OR “artificial intelligence” OR “deep learning” OR “neural network” OR “explainable AI” OR XAI) AND (“heart rate variability” OR HRV OR psychophysiol * OR “decision making” OR “executive function” OR “emotion regulation” OR “mental toughness” OR “cognitive performance” OR wearable OR biosensor OR cortisol))
Scopus	TITLE-ABS-KEY((soccer OR football) AND (“machine learning” OR “artificial intelligence” OR “deep learning” OR “neural network” OR “explainable AI” OR XAI) AND (“heart rate variability” OR HRV OR psychophysiol * OR “decision making” OR “executive function” OR “emotion regulation” OR “mental toughness” OR “cognitive performance” OR wearable OR biosensor OR cortisol))
Web of Science	TS = ((soccer OR football) AND (“machine learning” OR “artificial intelligence” OR “deep learning” OR “neural network” OR “explainable AI” OR XAI) AND (“heart rate variability” OR HRV OR psychophysiol * OR “decision making” OR “executive function” OR “emotion regulation” OR “mental toughness” OR “cognitive performance” OR wearable OR biosensor OR cortisol))
SPORTDiscus	(soccer OR football) AND (“machine learning” OR “artificial intelligence” OR “deep learning” OR “neural network” OR “explainable AI” OR XAI) AND (HRV OR psychophysiology OR “decision making” OR “executive function” OR “emotion regulation” OR “mental toughness” OR “cognitive performance” OR wearable OR biosensor OR cortisol)
IEEE Xplore	(soccer OR football) AND (“machine learning” OR “artificial intelligence” OR “deep learning” OR “neural network” OR “explainable AI” OR XAI) AND (“heart rate variability” OR HRV OR psychophysiology OR “decision making” OR “executive function” OR “emotion regulation” OR “mental toughness” OR “cognitive performance” OR wearable OR biosensor OR cortisol)
PsycINFO	(soccer OR football) AND (“machine learning” OR “artificial intelligence” OR “deep learning” OR “neural network” OR “explainable AI” OR XAI) AND (“decision making” OR “executive function” OR “emotion regulation” OR “mental toughness” OR psychophysiology OR HRV OR “cognitive performance” OR wearable OR biosensor OR cortisol)

* denotes the truncation symbol used in the search strategy to retrieve terms beginning with “psychophysiol” (e.g., psycholphysiology and psychophysiological).

**Table 3:** Distribution of the 36 included sources across thematic domains, with principal methods and key findings.

Domain	n	Primary AI/Methods	Psychological/Psychophysiological Constructs	Key Translational Findings
AI and ML in soccer: physical–tactical modeling	8	Decision trees, XGBoost, ANN, LSTM, random forest, explainable AI (SHAP)	Minimal; wellness indices in some load models [4]; no systematic psychological constructs	ML predicts match outcomes, injury risk, and physical performance at internal-validation accuracy above 66%; psychological inputs remain the missing layer [3,4,5]
Psychophysiology and psychological constructs in soccer	8	Salivary cortisol, HRV, EEG, eye-tracking; cognitive test batteries	Executive function, decision-making, attentional control, emotional regulation, resilience, psychological profile (Big Five and EF composite)	Elite players show stronger EF profiles than sub-elite players [2,9,10]; psychophysiological stress changes EF under game-realistic conditions [11]
HRV monitoring and training load in soccer	6	RMSSD, LnRMSSD, LF/HF ratio, nocturnal HRV via actigraphy; morning HRV via PPG wearables	Autonomic readiness, recovery status, psychological stress markers (pre-competition cortisol–HRV coupling)	Morning HRV is ecologically feasible; nocturnal HRV is more sensitive to load; individual variability requires athlete-level interpretation [12,13,14]
Wearable sensors and biosensors for athlete monitoring	3	GPS, IMU, EDA, PPG, ECG, salivary biosensors, smart textiles	Internal workload, autonomic arousal, biochemical stress markers (cortisol, lactate), alertness proxies	Multi-modal wearable fusion enables joint internal–external workload profiling; EDA and cortisol sensing remain immature for field use [20,21,22]
AI for psychophysiological mental-state monitoring	5	ML applied to HRV, EDA, accelerometry, facial expression, multi-modal fusion; ANN, SVM, gradient boosting	Stress, anxiety, depression, mental readiness, emotional-state classification	Multi-modal biosensor and AI reaches internal-validation accuracy above 85% for stress classification in athletes; soccer-specific validation is absent [15,16,23,24]
Explainable AI (XAI) in sports science and soccer	2	SHAP values, LIME, Grad-CAM, individual conditional expectation plots	Largely absent; physical and tactical variables dominate input features	XAI methods are emerging but rarely validated with coaches; psychological constructs are absent from XAI soccer models [7,25,26]
Biofeedback, neurofeedback, and self-regulation	4	HRV biofeedback, EEG neurofeedback, respiratory biofeedback	Anxiety, arousal regulation, attentional control, cognitive performance, emotional self-regulation	HRV biofeedback produces moderate effects for anxiety and performance [27]; neurofeedback improves reaction time and attention [28]; no soccer-specific AI integration

Abbreviations used in Table 3: ANN, artificial neural network; ECG, electrocardiography; EDA, electrodermal activity; EEG, electroencephalography; Grad-CAM, gradient-weighted class activation mapping; GPS, global positioning system; IMU, inertial measurement unit; LF/HF, low-frequency-to-high-frequency power ratio; LnRMSSD, natural logarithm of the root mean square of successive differences; LSTM, long short-term memory; PPG, photoplethysmography; RMSSD, root mean square of successive differences; SHAP, SHapley Additive exPlanations; SVM, support vector machine; XAI, explainable artificial intelligence.

## Data Availability

The completed PRISMA-ScR checklist (Supplementary Material S1) and the full charting table of included sources (Supplementary Material S2) accompany this article. The screening log is available from the corresponding author on reasonable request.

## Supplementary Materials

The following supporting information can be downloaded at https://www.mdpi.com/article/10.3390/sports14080360/s1: Supplementary Material S1: Completed PRISMAScR checklist; Supplementary Material S2: Characteristics and classification of the included sources.

## Abbreviations

AI: artificial intelligence; ANN: artificial neural network; DHEA: dehydroepiandrosterone; ECG: electrocardiography; EDA: electrodermal activity; EEG: electroencephalography; EF: executive function; ES: effect size; GPS: global positioning system; Grad-CAM: gradient-weighted class activation mapping; HRV: heart rate variability; IMU: inertial measurement unit; JBI: Joanna Briggs Institute; LF/HF: low-frequency-to-high-frequency power ratio; LIME: local interpretable model-agnostic explanations; LnRMSSD: natural logarithm of the root mean square of successive differences; LSTM: long short-term memory; ML: machine learning; PCC: Population–Concept–Context; PPG: photoplethysmography; PRISMA-ScR: Preferred Reporting Items for Systematic reviews and Meta-Analyses extension for Scoping Reviews; RMSSD: root mean square of successive differences; SHAP: SHapley Additive exPlanations; SVM: support vector machine; XAI: explainable artificial intelligence; XGBoost: extreme gradient boosting.
