# Supplementary material for: Artificial Intelligence and Psychophysiological Monitoring for Integrated Performance Modeling in Elite Soccer: A Scoping Review of Applications, Evidence Gaps, and Translational Challenges
Source: Sports (Basel). 2026 Aug 19;14(8):360. doi: 10.3390/sports14080360 (PMC13517148; doi:10.3390/sports14080360)
Supplement: Supplementary file 1 [file sports-14-00360-s001.zip › Supplementary_S1_PRISMA-ScR_Checklist.pdf]

Supplementary Material S1.
Completed PRISMA-ScR checklist (Tricco et al., 2018).

Item	Checklist item	Reported in (this manuscript)	Section
TITLE			
1	Title.	Identifies the report as a scoping review.	Title
ABSTRACT			
2	Structured summary.	Structured abstract with Background, Aim, Methods, Results, Conclusions; registration stated.	Abstract
INTRODUCTION			
3	Rationale.	Rationale for an integrated AI–psychophysiology review and the literature gap.	Section 1
4	Objectives.	Four objectives and four explicit research questions (RQ1–RQ4) mapped to results sections.	Section 1
METHODS			
5	Protocol and registration.	Prospective OSF registration (https://osf.io/ad957/); registration identifier and date stated; PRISMA-ScR followed.	Section 2.1
6	Eligibility criteria.	PCC framework; inclusion of both primary studies and evidence syntheses justified.	Section 2.2; Table 1
7	Information sources.	Six databases plus reference-list hand-searching; search period January 2000 to March 2026.	Section 2.3
8	Search.	Full harmonized search strings for all databases; shared concept blocks; platform syntax adapted.	Section 2.3; Table 2
9	Selection of sources of evidence.	Covidence; two independent reviewers; kappa = 0.82; consensus and third-reviewer arbitration.	Section 2.4
10	Data charting process.	Standardized form piloted on eight sources; independent double charting; discrepancies resolved by a third reviewer.	Section 2.5
11	Data items.	Listed charted variables (author, year, country, evidence type, design, population, AI/ML method, constructs, wearables, findings, limitations).	Section 2.5
12	Critical appraisal of individual sources.	Not performed, consistent with JBI scoping methodology; stated explicitly.	Section 2.1; 2.5
13	Synthesis of results.	Descriptive and thematic synthesis across four domains; evidence weighted by type.	Section 2.5
RESULTS			
14	Selection of sources of evidence.	Numbers screened, assessed, and included with exclusion reasons.	Section 3.1; Figure 1
15	Characteristics of sources of evidence.	Year, country, and design distribution; full characteristics in Supplementary S2.	Section 3.2; Table 3; Suppl. S2
16	Critical appraisal within sources.	Not applicable (scoping review).	Section 2.1
17	Results of individual sources of evidence.	Findings reported by domain, with primary studies and reviews labeled.	Sections 3.3–3.6; Suppl. S2
18	Synthesis of results.	Thematic synthesis and three translational gaps.	Sections 3.3–3.6
DISCUSSION			
19	Summary of evidence.	Summary against the objectives and research questions.	Section 4

Item	Checklist item	Reported in (this manuscript)	Section
20	Limitations.	Language bias, heterogeneity, projective elements, and date boundary discussed.	Section 4.5
21	Conclusions.	Overall interpretation and research priorities.	Section 5; 4.4
FUNDING			
22	Funding.	Funding and role of funder stated.	Funding statement

Note. Section numbers refer to the revised manuscript. Item numbering follows the 22-item PRISMA-ScR checklist.
