# Supplementary material for: Artificial Intelligence and Psychophysiological Monitoring for Integrated Performance Modeling in Elite Soccer: A Scoping Review of Applications, Evidence Gaps, and Translational Challenges
Source: Sports (Basel). 2026 Aug 19;14(8):360. doi: 10.3390/sports14080360 (PMC13517148; doi:10.3390/sports14080360)
Supplement: Supplementary file 1 [file sports-14-00360-s001.zip › Supplementary_S2_Included_Studies.pdf]

Supplementary Material S2.

Characteristics and classification of the included sources.

Each source is classified by evidence type and by its role in the review. “Core” denotes soccer-specific evidence forming the primary evidence base. “Contextual” denotes feasibility evidence from adjacent populations admitted under the PCC translatability clause. Quantitative claims are attributed to primary studies; reviews are charted at the synthesis level. The charted evidence base comprises 36 sources. Three further references (items 28, 33, and 36) are methodological or foundational works cited for framing only and are not counted in the 36-source corpus. All counts reported in Section 3.2, Table 3, and the PRISMA flow diagram derive from these 36 sources.

#	Source	Evidence type / role in review	Population / sample	AI / ML methods	Psychophysiological / psychological variables	Key findings (and main limitation)
1	Addleman et al. (2024)	Narrative review / background	S&C athletes (review)	None	HRV (RMSSD), readiness	HRV-guided readiness and recovery monitoring in strength and conditioning. Limitation: narrative scope, no pooled effect.
2	Afonso et al. (2025)	Primary study / core	Sub-elite youth football players	Supervised ML	Physical, technical, tactical, maturational, recovery	Multidimensional data fusion outperformed single-domain models for recovery prediction. Limitation: sub-elite sample, no psychological inputs.
3	Alzahrani & Ullah (2024)	Review / contextual	Sports performance (review)	Analytics on wearable data	Biomechanical and health parameters	Wearable biomechanical analytics support precision health monitoring. Limitation: not soccer-specific, broad scope.
4	Badicu & Silva (2025)	Primary/applied / contextual	Athletes (mental-state focus)	Machine learning	Mental states, emotional state	ML applied to monitoring athletes' mental states; supports psychological-state classification. Limitation: not soccer-specific.
5	Bonetti et al. (2025)	Primary study / core	328 participants incl. 204 elite players (Brazil, Sweden)	Artificial neural networks, SVM	Executive function, cognitive flexibility, Big Five personality	Decoded the elite player psychological profile (higher cognitive flexibility, conscientiousness, openness). Limitation: cross-sectional, off-field laboratory assessment.
6	Botelho et al. (2022)	Primary study / core	24 elite female soccer players (26.4 ± 3.7 y)	Correlational analysis	Cortisol, testosterone, HRV (LF/HF), mood states	Cortisol related to mood and HRV; starter vs non-starter affective differences. Limitation: small sample, preseason only.
7	Chen et al. (2025)	Systematic review / contextual	Exercise and mental-health studies	ML on physiological signals	HRV, EDA, respiration, psychological state	HRV, EDA, and respiration are most informative for acute psychological-state change. Limitation: not soccer-specific.
8	Elstak et al. (2024)	Systematic review / core	Football-codes AI studies	Deep learning, general AI	Largely physical/tactical	Raised the opaque-model problem; framed explainability as an ethical obligation. Limitation: review-level, no psychological focus.
9	Esco et al. (2026)	Narrative review / background	Athletes (review)	None	HRV via mobile devices	Mobile HRV is feasible for monitoring adaptation and recovery. Limitation: narrative, device-validation heterogeneity.
10	Forcher et al. (2024)	Primary study / core	Elite soccer (tracking data)	Gradient boosting + SHAP (XAI)	None (tactical inputs)	Identified tactical signatures of defensive success with high ecological validity; most mature match-data XAI example. Limitation: no psychophysiological inputs.
11	García-Ortega et al. (2023)	Meta-analysis / core	Competitive soccer players	Meta-analytic synthesis	HRV (time/frequency domain)	Group effect small and non-significant (ES = 0.143); larger for females ($d = 0.275$, $p = 0.027$) and professionals ($d = 0.302$, $p = 0.007$). Limitation: heterogeneity across protocols.
12	Habekost et al. (2024)	Theoretical model / core	Elite soccer (conceptual)	None	Executive function, perception-action loop	Proposed a three-stage cognitive model (situational assessment, action selection, outcome evaluation). Limitation: conceptual, not empirically tested here.

#	Source	Evidence type / role in review	Population / sample	AI / ML methods	Psychophysiological / psychological variables	Key findings (and main limitation)
13	Haugan et al. (2025)	Scoping review (15 studies) / core	Soccer players	None	Executive function, game intelligence	EF positively associated with elite performance; ecological validity of lab tests is a persistent limitation.
14	Ivarsson et al. (2020)	Systematic review/meta / core	Football players	Meta-analytic synthesis	Decision-making, hope for success, task orientation	Psychological factors show small but significant prospective links with future performance. Limitation: small effect sizes.
15	Jianjun et al. (2025)	Primary study / contextual	480 athletes, multiple sports	Gradient boosting, neural networks	Mental toughness, athlete engagement, team cohesion; HRV, VO2, muscle activation	Multimodal physiological + psychological fusion reached R = 0.90 vs R = 0.77 for unidimensional models. Limitation: multi-sport, not soccer; needs external validation.
16	Kabeer Dilshith et al. (2026)	Opinion paper / core (conceptual)	Sports psychology (opinion)	None	Stress appraisal, biopsychosocial regulation	Reframed stress as a multisystem regulation process needing simultaneous physiological, psychological, contextual assessment. Used for framing only.
17	Kargarandehkordi et al. (2025)	Systematic review (48 studies) / contextual	Mental-health monitoring studies	ML on biosensor data	Stress, depression, anxiety; HRV, EDA, accelerometry	Multimodal sensor fusion outperformed single-signal models for psychological-state classification. Limitation: not soccer-specific.
18	Knöbel et al. (2024)	Primary study (RCT) / core	92 elite youth players (M = 15.2 y)	Experimental (stress induction)	Inhibition, cognitive flexibility; cortisol, HRV, subjective stress	Successful stress induction; no group interaction with cognition, but descriptive EF improvement and lower RT variability under stress. Limitation: youth sample, lab-field gap.
19	Kranzinger et al. (2026)	Scoping review (19 studies) / core	Sports-science XAI studies	SHAP, LIME, Grad-CAM	Largely absent (physical/tactical inputs)	SHAP dominant; psychological constructs absent; lack of domain-expert validation is the critical gap.
20	Lehrer et al. (2020)	Systematic review/meta / background	Mixed populations	Meta-analytic synthesis	HRV biofeedback; anxiety, performance	Small-to-moderate effects, largest for anxiety and athletic/artistic performance. Limitation: heterogeneous populations.
21	Lehrer & Gevirtz (2014)	Review (mechanistic) / background	General (review)	None	HRV biofeedback mechanisms	Explains how and why HRV biofeedback works (baroreflex, vagal). Limitation: mechanistic, non-soccer.
22	Li et al. (2025)	Primary study / contextual	National-level athletes	Binary logistic regression	Pre-sleep HRV; chronic insomnia	Pre-sleep HRV predicted insomnia with 96% accuracy in one sample. Limitation: single sample, no external validation.
23	Lipka et al. (2025)	Systematic review (19 studies) / core	Soccer players	None	HRV (RMSSD); overtraining, fatigue, recovery	RMSSD linked to overtraining symptoms, psychological fatigue, and recovery. Limitation: review-level heterogeneity.
24	Mirto et al. (2024)	Scoping review (25 studies) / core	Professional/semi-professional soccer	None	Morning vagally mediated HRV	Ultra-short morning orthostatic HRV is ecologically feasible for readiness. Limitation: protocol heterogeneity.
25	Moya et al. (2025)	Systematic review (172 papers) / core	Professional soccer	Supervised + deep learning	Largely physical/event data	Supervised and deep learning dominate; psychological/psychophysiological inputs in <8% of models.
26	Munoz-Macho et al. (2024)	Scoping review (32 studies) / core	Elite team sports (67% soccer)	Tree-based, general AI	Mostly physical/healthcare	Soccer is the most-studied sport; healthcare slightly outnumbers performance applications.
27	Nassis et al. (2023)	Review / core	Soccer (injury focus)	ML (general)	Injury risk inputs	ML applications in soccer with emphasis on injury risk. Limitation: narrow outcome focus.
28	Peters et al. (2020)	Method reference (not in 36-source corpus)	JBI scoping methodology	None	None	Provided the JBI methodology and PCC framework used here (method reference).

#	Source	Evidence type / role in review	Population / sample	AI / ML methods	Psychophysiological / psychological variables	Key findings (and main limitation)
29	Rico-González et al. (2023)	Systematic review (32 studies) / core	Soccer players	Decision trees, ML	Minimal (wellness indices)	Decision trees most common; injury accuracy >66% (internal validation). Limitation: external validation rarely reported.
30	Sartor et al. (2025)	Review / core (conceptual)	Sport psychology and talent ID	AI (general)	Psychological constructs, talent markers	Psychological constructs are underrepresented in AI talent and performance models. Used for framing.
31	Seshadri et al. (2019a)	Review / background	Athletes (review)	None	Physiological and biochemical profile; wearables	Wearable sensors for physiological/biochemical profiling. Limitation: device maturity varies.
32	Seshadri et al. (2019b)	Review / background	Athletes (review)	None	Internal/external workload; wearables	Wearable sensors for internal and external workload monitoring. Limitation: non-soccer-specific.
33	Stølen et al. (2005)	Foundational context (not in 36-source corpus)	Soccer (physiology)	None	Physical/physiological parameters	Foundational physiology of soccer; predates wearable/ML era. Context reference.
34	Teixeira et al. (2025)	Systematic review / core	Soccer (tactical behavior)	AI (general)	Tactical/collective dynamics	AI maps tactical behavior and collective dynamics; calls for standardized data protocols.
35	Tosti et al. (2024)	Systematic review / contextual	Athletes (neurofeedback)	EEG neurofeedback	Reaction time, attention, anxiety, emotion regulation	Neurofeedback improves reaction time and attentional control. Limitation: not soccer-specific, protocol variability.
36	Tricco et al. (2018)	Method reference (not in 36-source corpus)	PRISMA-ScR	None	None	Provided the PRISMA-ScR reporting checklist used here (method reference).
37	Wang et al. (2026)	Bibliometric analysis / core	HRV sports science 2010–2025	ML-guided HRV (mapped)	HRV; ML interpretation	Identified ML-guided HRV interpretation as an emerging, under-explored frontier.
38	Zhang et al. (2025)	Bayesian meta-analysis / contextual	Athletes (biofeedback)	Bayesian meta-analysis	HRV biofeedback; anxiety, performance	HRV biofeedback produces moderate effects for anxiety and performance. Limitation: not soccer-specific.
39	Zhu et al. (2024)	Systematic review/meta / core	Team-sport athletes	Meta-analytic synthesis	Perceptual-cognitive training; anticipation, decision-making	Larger lab effects (ES = 1.51) than real-game transfer (ES = 0.65), underscoring the ecological-validity gap.
